# Oxidative stress in rat brain during experimental status epilepticus: effect of antioxidants

**DOI:** 10.3389/fphar.2023.1233184

**Published:** 2023-09-12

**Authors:** Marius Fuchs, Christian Viel, Alina Lehto, Helene Lau, Jochen Klein

**Affiliations:** Institute of Pharmacology and Clinical Pharmacy, College of Pharmacy, Goethe University, Frankfurt am Main, Germany

**Keywords:** ascorbic acid, α-tocopherol, coenzyme Q10, resveratrol, ebselen, isoprostanes

## Abstract

Antioxidants have been proposed as a treatment for diseases of the central nervous system. However, few studies actually studied their effects in the brain. To test central actions of antioxidants, we used the lithium–pilocarpine (Li-Pilo) model of status epilepticus (SE) in the rat in which seizures are accompanied by significant oxidative stress. We used *in vivo* microdialysis to determine isoprostane levels during SE in real time and brain homogenates for other measures of oxidative stress. Six different antioxidants were tested in acute and preventive experiments (vitamin C, vitamin E, ebselen, resveratrol, n-tert-butyl-α-phenylnitrone and coenzyme Q10). None of the antioxidants had an effect when given acutely during SE. In contrast, when antioxidants were given for 3 days prior to seizure induction, vitamins C and E reduced isoprostane formation by 58% and 65%, respectively. Pretreatment with the other antioxidants was ineffective. In brain homogenates prepared after 90 min of seizures, SE decreased the ratio of reduced vs. oxidized glutathione (GSH/GSSG ratio) from 60.8 to 7.50 and caused a twofold increase of 8-hydroxy-2′-deoxyguanosine (8-OHdG) levels and protein carbonyls. Pretreatment with vitamin C or vitamin E mitigated these effects and increased the GSH/GSSG ratio to 23.9 and 28.3, respectively. Again, the other antioxidants were not effective. We conclude that preventive treatment with vitamin C or vitamin E ameliorates seizure-induced oxidative damage in the brain. Several well-studied antioxidants were inactive, possibly due to limited brain permeability or a lack of chain-breaking antioxidant activity in hydrophilic compounds.

## 1 Introduction

Reactive oxygen species (ROS) damage the cell in many ways, e.g., by oxidation of proteins or nucleic acids (mutagenic effect) or by oxidation of unsaturated fatty acids in cell membranes (lipid peroxidation) (Frijhoff et al., 2015; Halliwell and Gutteridge, 2015). ROS are detoxified endogenously by various mechanisms, enzymatically by superoxide dismutase or glutathione peroxidase and by endogenous low-molecular-weight antioxidants, e.g., vitamins C and E. In healthy cells there is a balance between formation and detoxification of ROS (“redox homeostasis”). If a state of increased ROS formation and/or diminished ROS detoxification is present, it is called “oxidative stress” (Sies, 2015). Oxidative stress occurs in inflammation, atherosclerosis and ischemia-reperfusion injury, contributes to the process of tumorigenesis, and is held (in part) responsible for the aging process (Frijhoff et al., 2015; Forman and Zhang, 2021). In addition, oxidative stress is an important feature of neurodegenerative and neurological diseases such as Alzheimer’s, Parkinson’s and epilepsy (Butterfield and Halliwell, 2019; Fabisiak and Patel, 2022). In the present work, we induced status epilepticus in rats to provoke oxidative stress in the brain. The formation of ROS and isoprostanes during seizures has been described before, e.g., after kainate injection (Patel et al., 2001) or after organophosphate administration (Zaja-Milatovic et al., 2008). We here use a microdialysis approach that allows continuous monitoring of oxidative stress in the brain (see below).

Substances that target oxidative stress have been repeatedly proposed as therapeutic options for neurodegenerative brain diseases including epilepsy (Lin et al., 2020; Forman and Zhang, 2021). These antioxidants include vitamins, plant polyphenols and synthetic agents. For the present study, we have selected six popular and often investigated antioxidants (Forman and Zhang, 2021): vitamin C (ascorbic acid), the natural antioxidant with hydrophilic characteristics, and vitamin E (α-tocopherol), the natural antioxidant with hydrophobic properties; as the selenium-containing ebselen, a glutathione peroxidas mimic; coenzyme Q10 (ubiquinone), an antioxidant present in mitochondria; N-tert-butyl-α-phenylnitrone (PBN), a nitrone radical scavenger; and resveratrol, an NRF2 activator.

However, most attempts to influence various diseases in humans with antioxidants have failed so far (Bjelakovic et al., 2012), and there is a particular shortage of treatment options for oxidative stress in the brain. A possible reason for this failure is the poor brain permeability of many antioxidants, as the blood-brain barrier is an effective barrier for drug penetration into the brain. To test central actions of antioxidants, we here used the lithium–pilocarpine (Li-Pilo) model of status epilepticus (SE) in the rat, in which pilocarpine, a directly acting cholinergic muscarinic agonist, is administered after pretreatment with lithium to initiate continuous seizures. This model is not only a popular model for SE and temporal epilepsy (Curia et al., 2008), the seizures are also accompanied by significant oxidative stress in the brain (Shin et al., 2011; Shekh-Ahmad et al., 2019; Lin et al., 2020; Fabisiak and Patel, 2022). Previous studies in our group showed a 20-fold increase of isoprostanes in rat hippocampus during seizures that were induced with Li-Pilo (Imran et al., 2015). Isoprostanes are considered the most specific indicators of oxidative stress, especially with respect to lipid peroxidation (Halliwell and Whiteman, 2004; Halliwell and Lee, 2010; Milne et al., 2015). In the present study, we used microdialysis and monitored the formation of isoprostanes in the hippocampus before, during and after seizures. We investigated the effects of preventive and acute treatment of six antioxidants: vitamin C, vitamin E, coenzyme Q10, N-tert-butyl-α-phenylnitrone (PBN), resveratrol, and ebselen. In addition to isoprostanes, we also measured GSH-GSSG ratios, 8-hydroxy-2′-deoxyguanosine (8-OHdG), protein carbonyls and malondialdehyde (MDA) in brain homogenates. The rationale of the study was to study antioxidant action in the brain by on-line monitoring, with the important corollary that oral administration of the antioxidants in an in vivo-model would also test their ability to reach effective concentrations in the brain to affect ROS formation.

## 2 Methods

### 2.1 Animals

Male Sprague Dawley rats (6 weeks old, 220–300 g) were obtained from Janvier laboratories (Le Genest-Saint-Isle, France) and housed in standard rodent cages (three rats per cage) in the departmental animal house facility. Animals had access to food (Altromin 1320; Lage, Germany) and water *ad libitum* and were kept under standard conditions (temperature 20°C–22°C, 50%–65% relative humidity; 17–20 air changes per hour) and on a 12-h light/dark cycle (07:00 a.m. to 07:00 p.m.). After at least 1 week of adaptation in the animal facility, rats were randomly assigned to study groups (8–10 per group) by using block randomisation (Latin square design). Based upon 3 animals per cage, rats were assigned to 3 groups in the following order: ABC, ACB, BAC, BCA, CAB, and CBA. In total, 132 animals were used. 117 experiments were successful (101 rats with SE and 16 controls). Five rats died during SE, ten rats had to be excluded because of blocked or leaking microdialysis probes. All animal procedures were carried out to minimize animal suffering in accordance with German and European law (EU directive 2010/63/EU), and the study was registered with the local authorities (RP Darmstadt; FR1023).

### 2.2 Treatments

**FIGURE 1:**
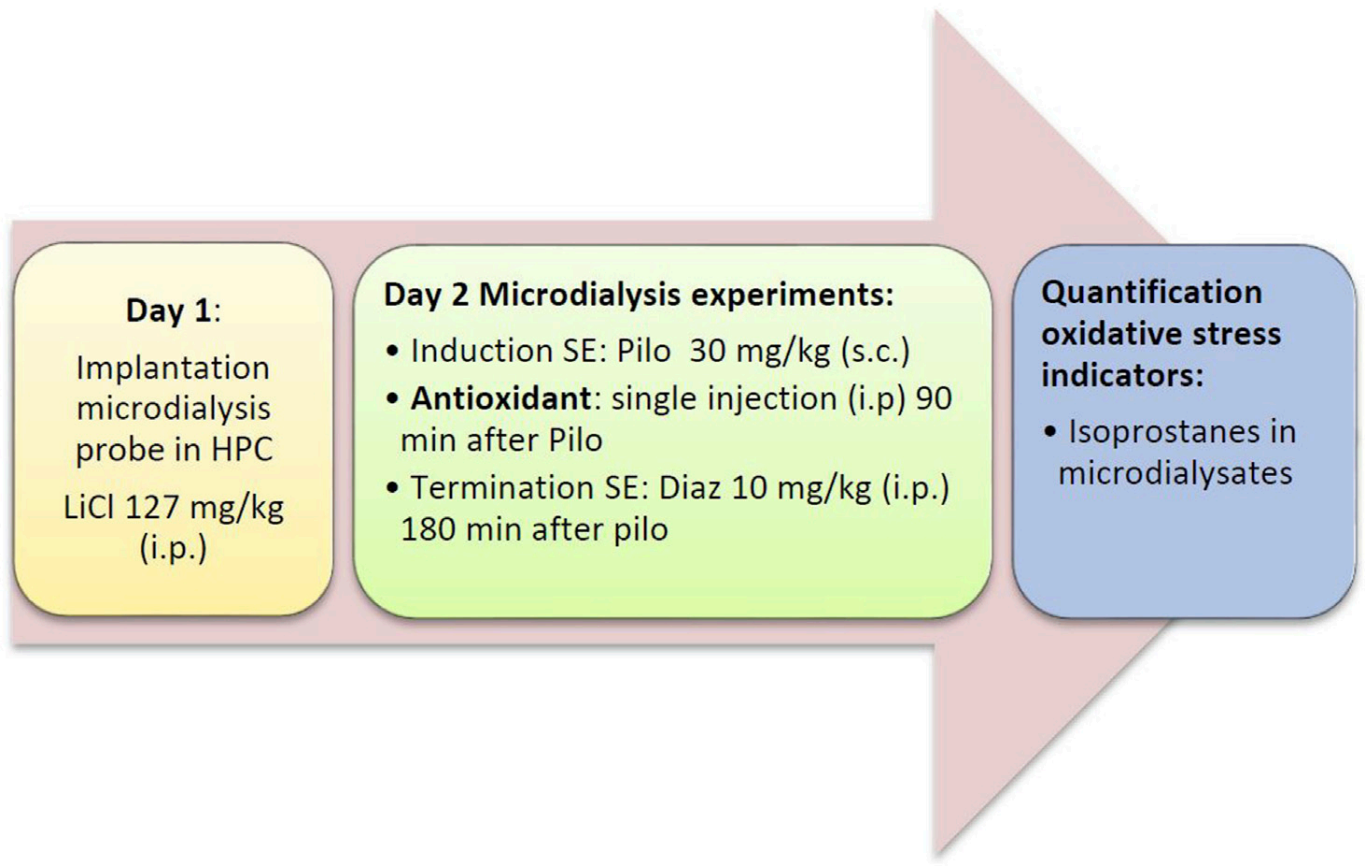
Flow diagram of the acute series of experiments. Diaz, diazepam; HPC, hippocampus; LiCl, lithium chloride; Pilo, pilocarpine; SE, status epilepticus.

Status epilepticus was induced by administration of lithium chloride (127 mg/kg i. p.) on the day of probe implantation and by injection of pilocarpine (30 mg/kg s.c.) on the day of the experiment (for details, see below). In addition, rats were treated with three endogenous antioxidants, vitamin C (L-ascorbic acid; Merck, Darmstadt, Deutschland), vitamin E (α-tocopherol; TCI, Tokyo, Japan), and coenzyme Q_10_ (Alfa Aesar, Ward Hill, United States), two synthetic antioxidants, ebselen (TCI, Tokyo, Japan), and N-tert-butyl-α-phenylnitrone (PBN; TCI, Tokyo, Japan), and one plant polyphenol, resveratrol (TCI, Tokyo, Japan). The control group received saline. There were two different series of experiments: In the acute series of experiments (Figure 1), antioxidant/saline was administered acutely (by i.p. injection) 90 min after pilocarpine injection. In the preventive series of experiments, antioxidant/saline was administered orally (by gavage) for three and a half days before inducing status epilepticus, i.e., on days 1–3, antioxidant/saline was administered in the morning and evening, and on day 4 (the status epilepticus experimental day) only in the morning. Table 1 below lists the antioxidants used, including corresponding doses.

**TABLE 1:** Doses of antioxidants and saline. PBN = n-tert-butyl-α-phenylnitrone.

Antioxidant/control	Acute treatment	Preventive treatment
Vitamin C	250 mg/kg	500 mg/kg
Vitamin E	100 mg/kg	200 mg/kg
Coenzyme Q10	100 mg/kg	200 mg/kg
PBN	100 mg/kg	200 mg/kg
Ebselen	10 mg/kg	20 mg/kg
Resveratrol	50 mg/kg	100 mg/kg
Saline	1 mL/kg	5 mL/kg

Vitamin C and PBN were dissolved in saline (B.Braun, Melsungen, Germany), vitamin E and coenzyme Q_10_ in corn oil (Carl Roth, Karlsruhe, Germany). For ebselen and resveratrol, a different vehicle was used in each of the acute and preventive series of experiments, namely 5% DMSO dissolved in saline for injection and a modified O/W Emulsion for oral gavage as described previously (Viel et al., 2021). Drugs and chemicals of general use were supplied by Merck (Darmstadt, DE) or Sigma (Munich, DE) at the highest purity available.

### 2.3 Microdialysis experiments

Prior to probe implantation rats were single housed in microdialysis cages in the experimental room. Before starting the surgery, a habituation period of at least 1 hour was allowed. Rats were weighed and anesthetized with isoflurane (induction dose 5%, maintenance dose 1,5%–2% v/v; Iso-Vet, Dechra Veterinary Products, Aulendorf, DE) in synthetic air (Praxair, Düsseldorf, Germany) and placed in a stereotaxic frame (Stoelting, Chicago, IL, United States). Y-shaped, concentric microdialysis probes (Polysulfone membrane FX CorDiax 600, Fresenius Medical Care, Bad Homburg, Germany) with a molecular weight cut-off of 30 kDa and an exchange area of 3.5 mm were manufactured as previously described (Lietsche et al., 2014). They were implanted in the right ventral hippocampus of fully anesthetized rats using the following coordinates (from bregma): AP −5.2 mm; L −5.2 mm; DV −7.0 mm (Paxinos and Watson, 1998). Bupivacaine (Jenapharm, Jena, Germany) was applied for long-lasting pain relief. After surgical implantation of probes, all rats received an injection of lithium chloride (3 mmol/kg i.p., equivalent to 127 mg/kg; Sigma, Munich, Germany). To prevent dehydration, all rats received 2 mL of Ringer-lactate solution (i.p.; B. Braun, Melsungen, Germany). After implantation, rats recovered overnight. A minimum of 18 h was kept between probe implantation and start of the experiment.

**FIGURE 2:**
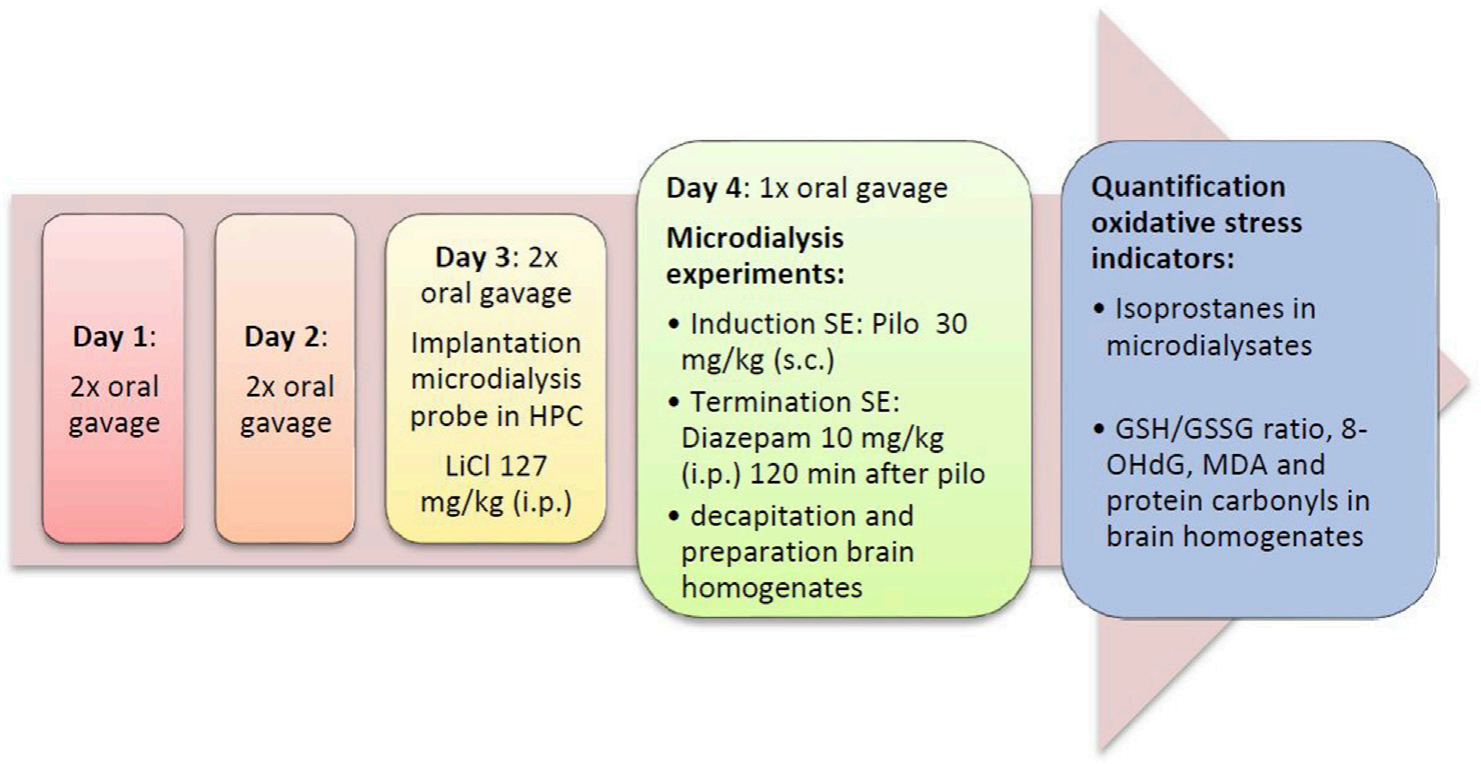
Flow diagram of the preventive series of experiments. Diaz, diazepam; GSH/GSSG ratio, ratio of reduced vs. oxidized gluthatione; HPC, hippocampus; LiCl, lithium chloride; SE, status epilepticus; MDA, malondialdehyde; Pilo, pilocarpine; 8-OHdG, 8-hydroxy-2′-deoxyguanosine.

On the following day, microdialysis was performed between 09:00 and 17:00 h. Rats were briefly restrained to connect the probe to the pump. The probes were perfused with artificial cerebrospinal fluid (aCSF; 147 mM NaCl, 4 mM KCl, 1.2 mM CaCl_2_, and 1.2 mM MgCl_2_; all VWR, Darmstadt, Germany) at a rate of 2 μL/min. The efflux from the microdialysis probes was collected in 30 min intervals. To prevent artificial formation of isoprostanes, dialysates were stored at −80°C under protective conditions (0.005% butylated hydroxytoluene). After 30 min of equilibrium between perfusion liquid and tissue, dialysates were collected for 90 min (3 samples) to determine baseline levels of analytes. Thereafter, pilocarpine (30 mg/kg s. c. in saline) was given to induce SE that developed within 30 min. 90 min after pilocarpine administration, i.e., during SE, the antioxidants were injected in the acute treatment groups (Figure 1). No drugs were given during SE in the preventive treatment group (Figure 2). Diazepam (10 mg/kg i.p.), a benzodiazepine that is first-line therapy for SE in humans (Chen and Wasterlain, 2006; Seinfeld et al., 2016), was given either 2 (preventive treatment) or 3 h (acute treatment) after pilocarpine administration to terminate seizures.

After the end of the microdialysis experiments, rats were decapitated under brief isoflurane anaesthesia. To confirm the implantation site on a random basis, some probes were perfused with the dye Fast Green (50 mM in aCSF; Sigma, Munich, Germany, F7258) prior to sacrifice. For the determination of oxidative stress indicators, brains were harvested directly after decapitation. The brain was kept on an ice-cooled Petri dish, and cerebellum and olfactory bulb were quickly removed and discarded. The hemispheres were separated, weighed and immediately frozen in liquid nitrogen. Brain hemispheres were stored at −80°C until further analysis.

### 2.4 Behavioral scoring

Rat behavior was scored every 5 min after pilocarpine injection according to a modified Racine scale (Racine, 1972). Six stages of seizure development were distinguished: Stage 0, no behavioral disturbance. Stage 1, piloerection, salivation, slight tremor, chromodacryorrhea and diarrhea (signs of muscarinic stimulation). Stage 2, stereotypical behavior, repeated chewing, rats are calm and stare into space. Stage 3, seizures in limbs, head bobbing, sniffing, but rats remain conscious. Stage 4, rats are unconscious with tremors, show no response to stimulus and lie immobile but have no convulsions. Stage 5, status epilepticus, typically with alternating rearing and lowering of the body every 30–60 s and unconsciousness (animal does not respond to external stimuli). Stage 6, tonic-clonic seizures, stretching spasms and clonic convulsions, typically with shortness of breath. Stage 6 was not observed in our experiments.

### 2.5 Quantification of isoprostanes in dialysates

Isoprostanes were determined by the 8-isoprostane express ELISA Kit manufactured by Cayman Chemical (Item-No. 516360, Cayman Chemical, Ann Arbor, United States). The assay is a competitive ELISA with a limit of detection of approximately 10 pg/mL. The kit was used according to the manufacturer’s protocol.

### 2.6 Analytical measurements in brain homogenates

Brain homogenates were prepared for the determination of the GSH/GSSG ratio, 8-OHdG, protein carbonyls and malondialdehyde (MDA). Brain hemispheres and PBS buffer pH 6.0 (10 mM) were mixed in a cooled Potter vessel in a ratio of 1:9 (hemisphere: buffer), immediately followed by homogenisation (15 hits at 1,500 rpm; Potter S, B. Braun, Melsungen, Germany). The slightly acidic pH prevents glutathione (GSH) from spontaneously oxidizing to glutathione disulfide (GSSG). For MDA quantification, brain homogenates were prepared with PBS buffer pH 7.4 in a ratio of 1:10 (hemisphere: buffer).

The GSH/GSSG ratio was determined using the GSH/GSSG ratio detection assay kit II (Item-No. ab205811, Abcam, Cambridge, United Kingdom). Reduced (GSH) and oxidized (GSSG) glutathione were quantified using the fluorescent indicator Thiol Green (Abcam), which forms adducts with thiol groups. The GSH/GSSG ratio was calculated from the concentrations of the two analytes. Prior to quantification, homogenates were centrifuged (12,000 g, 15 min, 4°C). The supernatants were deproteinized with trichloroacetic acid (TCA) in a ratio of 1:10 (supernatant: TCA). After a second centrifugation step (12,000 g, 5 min, 4°C) supernatants were neutralized and analysed according to the manufacturer’s protocol. The limit of detection was about 10 nM for GSH and GSSG, respectively.

For the determination of 8-OHdG, homogenates were centrifuged (5,000 g, 5 min, 4°C) and supernatants were used for analysis. 8-OHdG was analysed using Rat 8-OHdG ELISA Kit (Item-No. RTFI01271, Assay Genie, Dublin, Ireland), a competitive peroxidase-based ELISA with a detection limit of 0.94 ng/mL. The kit was used according to the manufacturer’s protocol.

Protein carbonyls were analysed using Protein Carbonyl Colorimetric Assay Kit (Item-No. 10005020, Cayman Chemical, Ann Arbor, United States). Quantification of protein carbonyls was based on the DNPH reaction. The detection limit was about 0.74 nmol/L. After centrifugation of homogenates (10,000 g, 15 min, 4°C) supernatants were used for analysis according to the manufacturer’s protocol.

Malondialdehyde (MDA) was quantified by a modified thiobarbituric acid (TBA) assay (Hall and Bosken, 2009). Since MDA is chemically unstable, the precursor 1,1,3,3-tetramethoxypropane (Merck, Darmstadt, Deutschland), a stable acetal form of MDA, was used to prepare a standard curve; 1 mol of tetramethoxypropane yields a 1 mol of MDA. Standards were freshly dissolved in ultrapure water at 10 nM–0.5 µM. The absorbance values were determined at a wavelength of 535 nm.

Protein concentrations were determined according to Bradford (Bradford, 1976), using albumin fraction V 96% as standard.

### 2.7 Data analysis and statistics

**FIGURE 3:**
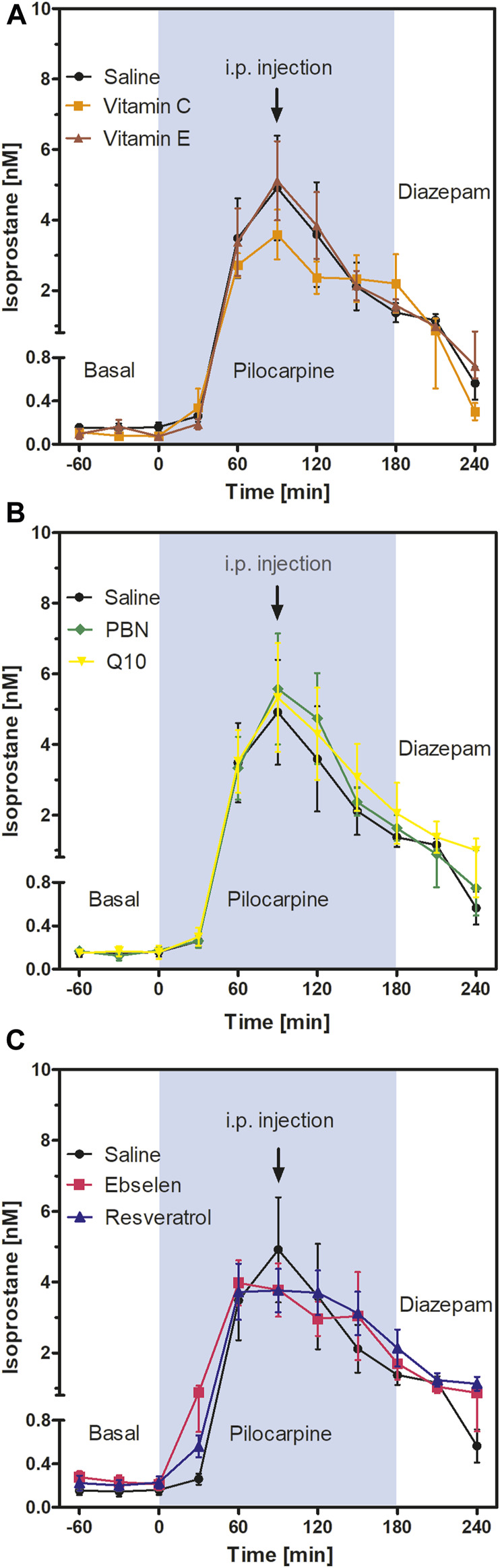
Extracellular isoprostane concentrations in the ventral hippocampus before SE (“Basal”), during SE (“Pilocarpine”, indicated as blue background) and after administration of diazepam (“Diazepam”) (acute series of experiments, *cf.* Figure 1). Antioxidant or saline were injected 90 min after pilocarpine administration. Data are presented as means ± SEM, given as absolute values. Treatments: **(A)** vitamin C (*n* = 9) and vitamin E (*n* = 6); **(B)** n-tert-butyl-α-phenylnitrone (PBN; *n* = 7) and coenzyme Q10 (Q10; *n* = 7); **(C)** ebselen (*n* = 6) and resveratrol (*n* = 7). Injections of saline (*n* = 8) served as controls. Statistics (two-way ANOVA with Bonferroni´s post-test): **(A)** F_2,20_ = 0.27; *p* = 0.77. **(B)** F_2,20_ = 0.14; *p* = 0.87. **(C)** F_2,18_ = 0.07; *p* = 0.93.

**FIGURE 4:**
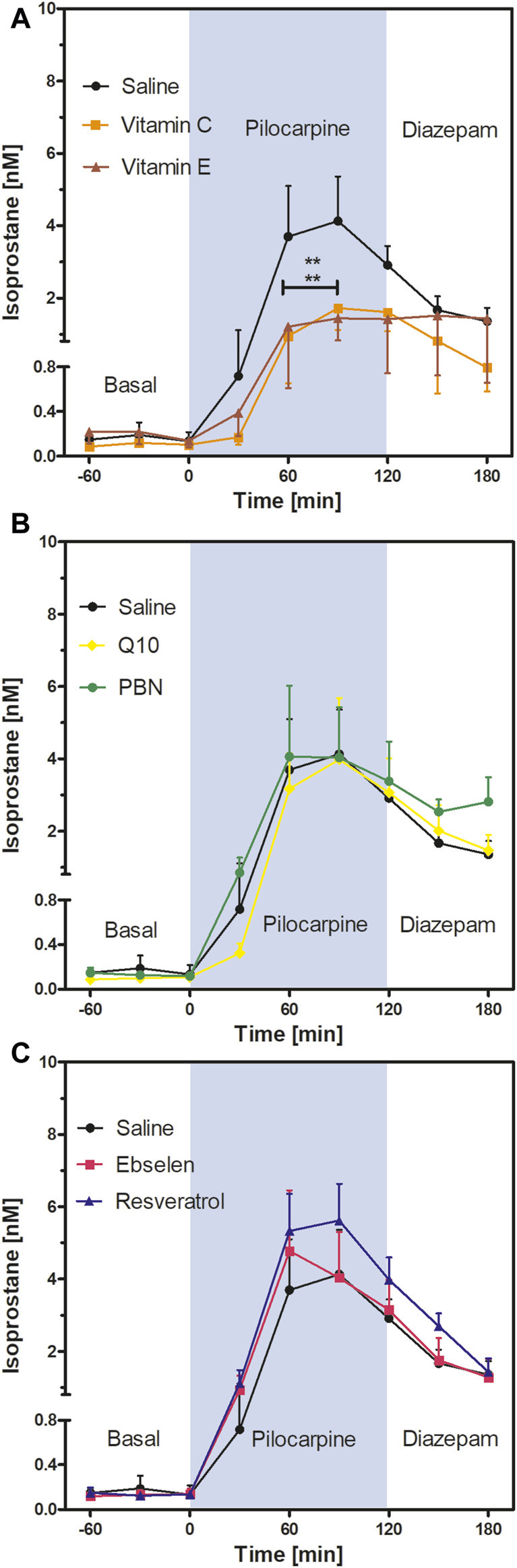
Extracellular isoprostane concentrations in the ventral hippocampus before SE (“Basal”), during SE (“Pilocarpine”, indicated as blue background) and after administration of diazepam (“Diazepam”) (preventive series of experiments, *cf.* Figure 2). Rats received seven dosages of antioxidant or saline every 12 hours prior to seizure induction. Data are presented as means ± SEM, given as absolute values. Treatments: **(A)** vitamin C (*n* = 8) and vitamin E (*n* = 7); **(B)** n-tert-butyl-α-phenylnitrone (PBN; *n* = 6) and coenzyme Q10 (Q10; *n* = 8); **(C)** ebselen (*n* = 7) and resveratrol (*n* = 8). Pretreatment with saline (*n* = 7) served as control. Statistics (two-way ANOVA with Bonferroni’s post-test): **p < 0.01 *versus* saline. **(A)** F_2,19_ = 2.23; *p* = 0.13. **(B)** F_2,18_ = 0.19; *p* = 0.83. **(C)** F_2,19_ = 0.58; *p* = 0.57.

**FIGURE 5:**
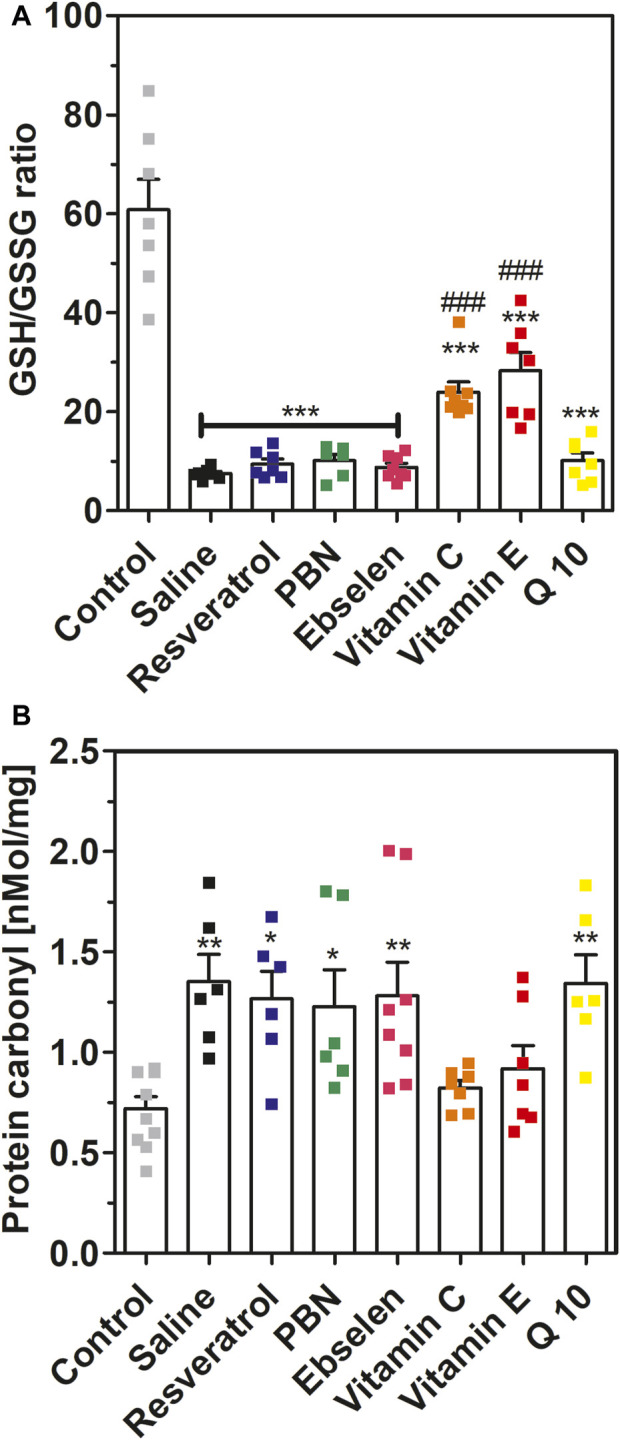
Glutathione ratio and protein carbonyls measured in brain homogenates after 2 h of SE. Rats received seven dosages of antioxidant or saline every 12 hours prior to seizure induction (preventive series of experiments, *cf.* Figure 2). SE was not induced in control animals. Data are presented as means ± SEM. Abbreviations: PBN, n-tert-butyl-α-phenylnitrone; Q10, coenzyme Q10. **(A)** Ratio of reduced vs. oxidized glutathione (GSG/GSSG ratio). Number of experiments as indicated (*n* = 6–8). Statistics (one-way ANOVA with Dunnett’s multiple comparison test): F_7.49_ = 28.66; *p* < 0.0001. ***, *p* < 0.001 vs. control. ###, *p* < 0.001 vs. saline. **(B)** Concentrations of protein carbonyls in brain homogenates. Data are presented as means ± SEM, normalised to protein. Number of experiments as indicated (*n* = 6–10). Statistics (one-way ANOVA with Dunnett’s multiple comparison test): F_7.48_ = 4.557; *p* < 0.001.; *, *p* < 0.05; **, *p* < 0.01 vs. control.

**FIGURE 6:**
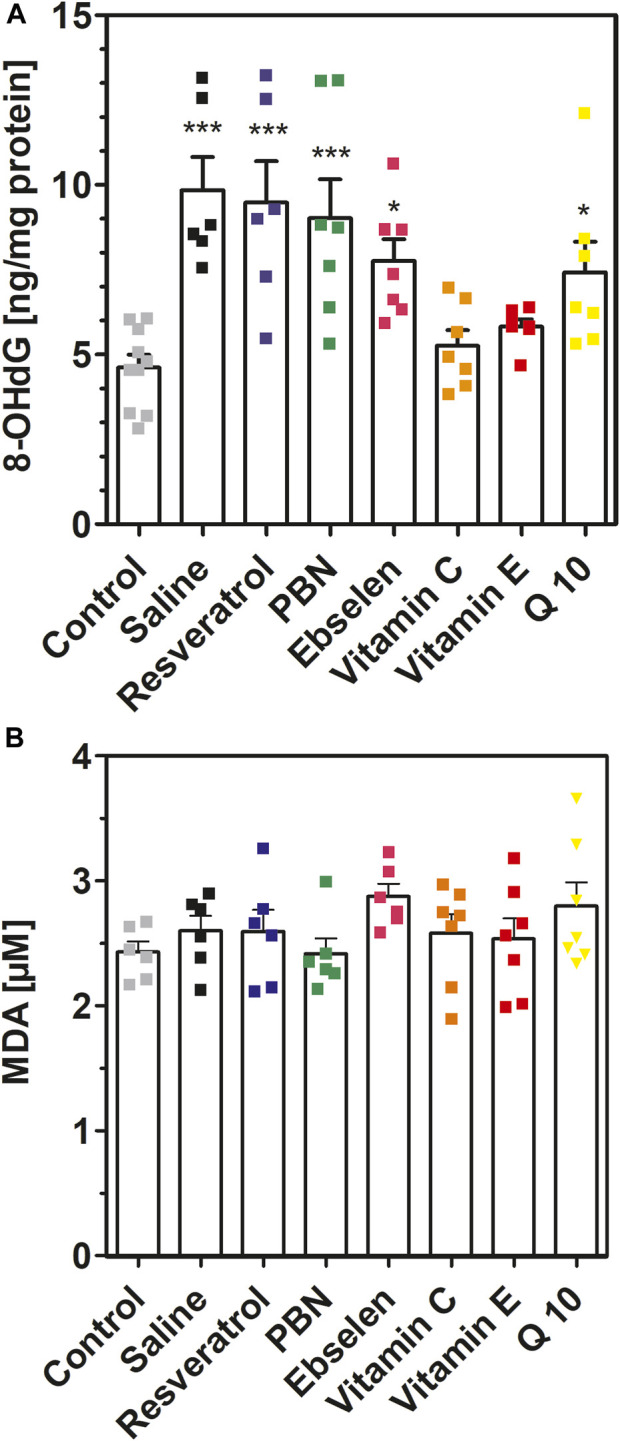
8-hydroxy-2′-deoxyguanosine and malondialdehyde measured in brain homogenates after 2 h of SE. Rats received seven dosages of antioxidant or saline every 12 hours prior to seizure induction (preventive series of experiments, *cf.* Figure 2). SE was not induced in control animals. Abbreviations: PBN, n-tert-butyl-α-phenylnitrone (PBN); Q10, coenzyme Q10. **(A)** Concentrations of 8-hydroxy-2′-deoxyguanosine (8-OHdG). Data are presented as means ± SEM, normalised to protein. Number of experiments as indicated (*n* = 6–10). Statistics (one-way ANOVA with Dunnett’s multiple comparison test): F_7.49_ = 7.073; *p* < 0.0001. *p < 0.05; ***p < 0.001 vs. control. **(B)** Concentrations of malondialdehyde (MDA) in brain homogenates. Data are presented as means ± SEM. Number of experiments as indicated (*n* = 6, 7). Statistics (one-way ANOVA): F_7,43_ = 1.175; *p* = 0.34.

This is an exploratory study using absolute isoprostane values and oxidative stress indicators in brain homogenates as outcomes. The study was not pre-registered. The experimenter was blinded to the animal groups during the measurements of oxidative stress indicators. If not indicated otherwise, data are presented as mean ± SEM of *N* animals. Normal distribution was tested using the Kolmogorov-Smirnov test. Potential outliers (>*2 SD*) were identified by the Grubbs test. Sample size was calculated by formula N = 2 SD^2^ × power index/delta^2^. From many years of experience with microdialysis, an SD of 20% was expected and a treatment effect of 25% was defined as goal of the study. The value for the power index (α = 0.05, two-sided; ß = 0.2; 80%) was taken from the book “Intuitive Statistics” by Harvey Motulsky (Oxford University Press, 1995). Time courses of isoprostane levels (Figures 3, 4) were compared using two-way analysis of variance for repeated measurements with Bonferroni’s post-test for multiple pairwise comparisons. One-way ANOVA followed by Dunnett’s multiple comparison test was used to compare oxidative stress indicators between eight groups (Figures 5, 6). Statistics were calculated with GraphPad Prism 5.03 software (GraphPad Software, La Jolla, CA, United States).

## 3 Results

### 3.1 Behavioral observation

Seizure severity was evaluated according to the modified Racine scale as described in Methods. Cholinergic signs (stage 1), such as piloerection, salivation and chromodacryorrhea, appeared immediately after injection of pilocarpine. Stage 2 was reached after 5–10 min (hypoactive animals, chewing, sniffing). Stage 3 signs (motoric responses as tremors and head bobbing, but no loss of consciousness) were frequent between 10 and 20 min after pilocarpine administration. Stage 3 went over to stage 4 (motoric responses with loss of consciousness), lasting for less than 5 min before animals developed status epilepticus (Stage 5) approx. 25–30 min after pilocarpine injection. SE is characterized by persistent rearing and lowering of the body and unconsciousness.

In our hands, neither acute injections of antioxidants (90 min after pilocarpine injection) nor oral pretreatment with antioxidants for 3 days prior to seizure induction affected seizure severity or duration. Exemplary data are shown in Supplementary Figures S1, S2. SE was successfully terminated by diazepam (10 mg/kg i. p.) 180 min (acute treatment series) or 120 min (preventive treatment series) after pilocarpine administration. Tonic-clonic seizures (stage 6) were never reached in our experiments.

### 3.2 Effects of acute antioxidant treatments on hippocampal isoprostane levels

To follow oxidative stress *in situ* in real time, we used microdialysis and monitored the formation of isoprostanes in hippocampus. Results are shown in Figure 3. Basal levels of isoprostanes in the ventral hippocampus of awake animals were 0.15 ± 0.01 nM (*n* = 50). Maximum isoprostane levels were achieved 90 min after pilocarpine injection and were 4.91 ± 1.49 nM (Figure 3; saline curve). This represents a highly significant, more than 30-fold increase in isoprostane formation during SE, confirming previous studies by our group (Imran et al., 2015).

To investigate acute effects of antioxidants, animals received saline or antioxidant by i.p. injection 90 min after pilocarpine administration, i.e., at the time point when isoprostane formation was highest. Two-way ANOVA of isoprostane curves between single injections of vitamin C, vitamin E, and saline (Figure 3A); PBN, Q10, and saline (Figure 3B); and ebselen, resveratrol, and saline (Figure 3C) revealed no significant differences between groups. This indicates that single administrations of antioxidants do not acutely affect oxidative stress in the brain. Administration of diazepam suppressed seizures and decreased levels of isoprostanes in every treatment group to a similar extent (Figure 3).

### 3.3 Effects of preventive antioxidant treatments on hippocampal isoprostane levels

To evaluate the preventive effect of antioxidants, we administered antioxidants or saline for 3 days, every 12 hours, prior to seizure induction. Basal extracellular isoprostane levels were quantified in each treatment group and are shown in Table 2. Notably, no significant difference was found between the basal levels of the saline group and the different treatment groups with antioxidants (one-way ANOVA F_6,146_ = 1.12, *p* = 0.36).

**TABLE 2:** Basal values of isoprostanes as determined by microdialysis. PBN = n-tert-butyl-α-phenylnitrone.

	Saline	Vitamin C	Vitamin E	PBN	Q10	Ebselen	Resveratrol
Isoprostanes [nM]	0.16 ± 0.05	0.10 ± 0.02	0.19 ± 0.05	0.13 ± 0.02	0.10 ± 0.01	0.13 ± 0.02	0.14 ± 0.02
Number of animals	7	8	7	6	8	7	8

Data are means ± SEM and were analysed by one-way ANOVA: F_6,146_ = 1.12, *p* = 0.36.

Comparison of the isoprostane time courses of the antioxidant- or saline-pretreated groups (two-way ANOVA) revealed a highly significant decrease in isoprostane levels in the vitamin C and vitamin E groups compared to saline (Figure 4A). Mean isoprostane levels at 90 min after pilocarpine injection were 4.13 ± 1.23 nM in the saline group *versus* 1.72 ± 0.60 nM in the vitamin C group and 1.44 ± 0.61 nM in the vitamin E group. In other words, the isoprostane maxima were reduced by 58% by vitamin C and by 65% by vitamin E, respectively, but they were still significantly higher than basal levels.

In contrast, the comparison of the isoprostane time courses after pretreatment with four other antioxidants did not reveal significant differences; neither PBN or Q10 (Figure 4B) nor ebselen or resveratrol (Figure 4C) affected isoprostane levels, indicating that pretreatment with these antioxidants did not affect oxidative stress in the brain. Termination of seizures by diazepam brought the levels of isoprostanes down in a similar, delayed manner in every treatment group.

### 3.4 Effects of preventive antioxidant treatments on oxidative stress indicators in brain homogenates

In addition to isoprostanes, we determined four further indicators of oxidative stress, namely, GSH/GSSG ratio, 8-OHdG, protein carbonyls and malondialdehyde, in brain homogenates. Status epilepticus decreased the ratio of reduced *versus* oxidized glutathione from 60.8 ± 6.12 (control, no SE) to 7.50 ± 0.41 (saline, SE) confirming severe oxidative stress in brain tissue during seizures (Figure 5A). This effect was highly significant (*p* < 0.001). Oral pretreatment with vitamin C or vitamin E mitigated this effect and stabilized GSH/GSSG ratios at 23.9 ± 2.11 and 28.3 ± 3.68, respectively (*p* < 0.001 vs. saline). In contrast, oral pretreatment with the other antioxidants had no significant effect on GSH/GSSG ratio in brain homogenates during seizures.

Furthermore, we investigated the effects of SE and antioxidant pretreatments on protein carbonyls (Figure 5B). Seizures increased protein carbonyls twofold from 0.72 ± 0.06 nmol/mg (control) to 1.35 ± 0.14 nMol/mg (saline). This effect was highly significant (*p* < 0.01). Pretreatment with ebselen, Q10, PBN or resveratrol also led to a nearly twofold increase of protein carbonyls during seizures. In agreement with the glutathione data, pretreatment with either vitamin C or vitamin E fully prevented the increase of protein carbonyls during SE (Figure 5B).

Similar findings were obtained for 8-hydroxy-2′-deoxyguanosine (8OHdG) (Figure 6A). Seizures resulted in a highly significant (*p* < 0.001) twofold increase of 8OHdG which increased from 4.62 ± 0.38 ng/mg (control) to 9.84 ± 0.98 ng/mg (SE). In accordance with our previous findings, pretreatment with either vitamin C or vitamin E prevented this increase. Pretreatment with PBN, Q10, ebselen and resveratrol was ineffective in reducing 8OHdG in brain homogenate.

Finally, we also quantified malondialdehyde in brain homogenates with a modified thiobarbituric acid assay (Figure 6B). In these experiments, no differences were visible when the means of eight experimental groups were compared (*p* = 0.34). Hence, the MDA method seems inappropriate for measuring oxidative stress in brain homogenates (see Discussion).

## 4 Discussion

### 4.1 Epileptic seizures and oxidative stress

Oxidative stress in the brain as a prominent pathological feature of acute seizures and status epilepticus was described in several human studies and animal models of SE (see Introduction). Likewise, ROS have been described as important pathological factors in other neurodegenerative diseases (Jiang et al., 2016; Salim, 2017). In agreement with previous studies, our study which used a Li-Pilo model of status epilepticus confirmed severe oxidative stress during seizures. We used microdialysis in the hippocampus to monitor the formation of isoprostanes and we measured the formation of 8-OHdG and protein carbonyls and a decrease of the GSH/GSSG ratio in brain homogenates. The advantage of the microdialysis method is that the formation and breakdown of the isoprostanes as oxidative stress markers can be followed *in vivo* in the brain of live animals and in real time.

The sources of ROS in the brain are not entirely clear. Major ROS sources in the brain include mitochondria and certain enzymes such as NADPH oxidase, xanthine oxidase and lipoxygenases (Nayernia et al., 2014; Angelova and Abramov, 2016). Conventionally, mitochondria have been assumed to be the major contributor to ROS production during seizures, especially complex I and III from the electron transport chain (Kovács et al., 2002; Turrens, 2003; Malinska et al., 2010). However, recent studies concluded that ROS in neurons are formed primarily by NADPH oxidase (NOX) in an NMDA receptor-dependent manner (Brennan et al., 2009; Kovac et al., 2014). We chose the lithium-pilocarpine model for this study in which seizures are initiated by continuous activation of muscarinic M1 receptors by pilocarpine, and the subsequent formation of inositol phosphates is strongly increased by previous treatment with lithium chloride (Curia et al., 2008). The massive increase of calcium in post-synaptic neurons causes hyperexcitability and probably a glutamatergic response as known from organophosphate-induced seizures. The present model was chosen because we observed a strong, seizure-induced oxidative stress response in previous studies focusing on cholinergic mechanisms and mitochondrial responses in the Li-Pilo model (Hillert et al., 2014; Imran et al., 2015).

We speculate that the seizure-induced ROS formation observed in this study may be due to glutamate-induced-excitotoxicity and activation of NADPH oxidase, but further work is required to substantiate this hypothesis. In our hands, SE caused damage to polyunsaturated lipids as arachidonic acid reflected in a 30-fold increase in isoprostanes. 2-4fold increases of isoprostanes in hippocampal or total brain homogenates were previously reported when kainic acid was used to induce seizures in rats (Patel et al., 2001) or mice (Zaja-Milatovic et al., 2008). Hence, either the Li-Pilo method of SE provokes more isoprostanes to be formed or the microdialysis method picks up more extensive increases of isoprostanes in the extracellular space than can be found in homogenates. SE-induced ROS formation also attacked proteins (twofold increase of protein carbonyl levels), DNA (twofold increase of 8-OHdG levels) and depleted endogenous antioxidants systems (highly significant decrease of GSH/GSSG ratio from ∼ 60 to 7.5). In contrast, MDA levels were not elevated after seizures, but this may be due to methodical limitations of the thiobarbituric acid (TBA) assay (Gutteridge and Quinlan, 1983; Halliwell and Gutteridge, 2015). Free MDA in biological system is low and more than 95% of MDA is formed by decomposition of lipid peroxides during heating in acid (Moore and Roberts, 1998). However, several compounds other than MDA react with TBA to give chromogens (Moore and Roberts, 1998), and this feature may have masked an initial difference in free MDA levels.

### 4.2 Effects of antioxidants on oxidative stress in the brain

Using the novel, microdialysis-coupled isoprostane assay, we tested the potential effects of six antioxidants on seizure severity and oxidative stress. Notably, significant antioxidant effects were observed in our study. We found a significant decrease of isoprostane levels in hippocampal dialysates after pretreatment with 7 doses of vitamins C or E prior to seizure induction. PBN, coenzyme Q10, ebselen and resveratrol were not effective in this paradigm. The significant effects of vitamin C or E pretreatmentwere corroborated by data from brain homogenates: the 3-day preventive treatment with vitamins C or E, respectively, fully prevented increases in 8-OHdG and protein carbonyls and strongly mitigitated the decrease of GSH/GSSG ratio. During acute treatment, no protective effects were seen. Our data suggest that single injections of vitamin C or vitamin E are not sufficient to reach effective levels in the brain whereas pretreatment with seven doses prior to seizure induction raised brain levels of these vitamins sufficiently to exert antioxidant effects. This finding is partly supported by other studies as discussed below.

In addition to its role as a cofactor in enzymatic reactions, vitamin C (ascorbic acid) has vital antioxidant properties, including the ability to scavenge hydroxyl radicals, and participates in the regeneration of vitamin E (May et al., 1998). In most cells, ascorbate is taken up by the highly selective sodium-vitamin C transporter (SVCT) 1 and 2; additionally, the oxidized form dehydroascorbate (DHA) can be absorbed through GLUT type transporters (Tsukaguchi et al., 1999). Gene expressions studies showed that SVCT2 is widely localized in neural tissues and choroid plexus, indicating that ascorbate enters the brain via cerebrospinal fluid compartments (Tsukaguchi et al., 1999). The prominent role of SVCT2 is evidenced by the fact that mice lacking SVCT2 show severely reduced levels of ascorbate in the cerebral cortex and increased levels of isoprostanes and MDA in the brain, which is associated with increased cell death (Harrison et al., 2010). There are also few publications in which ascorbate was given prior to high-dose pilocarpine and oxidative stress was determined six to 24 h afterwards in brain homogenates. Ascorbate reduced MDA levels (measured by the TBARS assay) and increased antioxidative enzymes (Santos et al., 2008; Dong et al., 2013), similar results were found with α-tocopherol (dos Santos et al., 2011; see below). As the severity of seizures was also reduced in these studies, the antioxidant could simply be a consequence of protection against seizures. Our present data, however, clearly show an antioxidative effect of ascorbate when given in a preventive fashion, and this effect occurred in the absence of seizure control. High doses of ascorbate were used in all studies, probably because basal levels of ascorbate are already very high (0.4 mM) under basal conditions (Miele and Fillenz, 1996).

Vitamin E (α–tocopherol) is the most important inhibitor of lipid peroxidation *in vivo* (Niki, 2014). Due to its lipid solubility, it concentrates in the interior of membranes and scavenges lipid peroxyl radicals, protecting in particular polyunsaturated fatty acids (PUFA) which are highly concentrated in the brain (Cobley et al., 2018). In a previous study, 2-week pretreatment with α–tocopherol (750 mg/kg/day) raised the brain levels of α-tocopherol more than 3-fold (Betti et al., 2011). When SE was induced by kainic acid, seizure severity remained unchanged but seizure-induced elevation of lipid peroxidation, measured as MDA in brain homogenate, and neuroinflammatory markers and neuronal cell death were attenuated (Betti et al., 2011; Ambrogini et al., 2018). In an organophosphate seizure model, a 3-day pretreatment with vitamin E (100 mg/kg/day) effectively prevented increases in isoprostanes and neuronal damage (Zaja-Milatovic et al., 2009). These studies, as well as our study, clearly show that pretreatment with α-tocopherol is required to exert antioxidant effects in the brain. The likely reason for this observation is, again, that α–tocopherol levels in the brain are high under control conditions. In fact, it is known that the brain is not affected by vitamin E deficiency (Spector, 1989).

The remaining four antioxidants were found to be inactive either after acute or preventive administration. Coenzyme Q10 (ubiquinone) is an essential component of the mitochondrial electron transport chain and a scavenger of alkyl peroxyl radicals (Bentinger et al., 2007). It was selected for this study because of previous suggestions of a neuroprotective action and an antioxidative action in the kainic acid-induced SE model (Baluchnejadmojarad and Roghani, 2013); the later activity was based on TBARS and nitrite assays in brain homogenates. PBN is a nitrone compound commonly used as a spin-trap molecule and radical scavenger. PBN is brain permeable (Chen et al., 1990; Cheng et al., 1993) and reduced neuronal degeneration in the Li-Pilo model of SE, especially in immature rats (Peterson et al., 2005; Kubová et al., 2018); however, oxidative stress was not measured in these studies. The lack of effect of PBN in our study is consistent with studies showing that PBN is not a potent chain-breaking antioxidant. An IC_50_ value of 20 mM was determined for PBN in tests for the inhibition of lipid peroxidation (Maples et al., 2001), and this indicates that PBN may be too hydrophilic to affect lipid peroxidation *in vivo*. In one study (Zaja-Milatovic et al., 2008), however, PBN was active in the brain when given by i. c.v. injection. This finding confirms that the low bioavailability of certain antioxidants in the brain contributes to their lack of effect.

Ebselen is a selenium compound that mimics the activity of glutathione peroxidase (GPx) (Parnham and Sies, 2013), inhibits lipoxygenases and activates the Nuclear factor erythroid 2-related factor 2 (Nrf2) system (Orian and Toppo, 2014). Ebselen is brain-permeable (Imai et al., 2001) and was suggested as a centrally acting antioxidant (Noguchi, 2016). However, ebselen did not exert any antioxidant effect in our study. We speculate that, similar to PBN, ebselen may be too hydrophilic to affect lipid peroxidation. Finally, we tested resveratrol, a polyphenolic compound that occurs naturally in grapes, red wine and peanuts. Resveratrol is a much-studied compound with anti-inflammatory and antioxidative properties (Kulkarni and Cantó, 2015). It is a known activator of the Nrf2 system (Forman and Zhang, 2021), and weak antioxidative activities (reduction of MDA levels) were reported in animal models of seizures (Gupta et al., 2002; Saha and Chakrabarti, 2014). As a consequence, resveratrol was suggested as treatment against epilepsy (Shetty, 2011). However, resveratrol showed no antioxidant effect in either acute or preventive treatment against SE. One reason for this failure may low bioavailability after oral administration. While resveratrol is sufficiently lipophilic to be well absorbed from the intestine, it is also rapidly metabolized (Walle, 2011) and apparently does not reach high levels in the brain (Juan et al., 2010). In addition, plant polyphenols such as resveratrol may also have pro-oxidative actions that prevent strong effects on oxidative stress (Halliwell, 2007).

### 4.3 Antioxidants and seizure severity

There are several reports in the literature suggesting anti-seizure-effects of antioxidants in animal models. For instance, a single injection of vitamin C (250 mg/kg) before seizure onset significantly reduced seizure latency and mortality in a pilocarpine seizure model in Wistar rats (Xavier et al., 2007). Also in Wistar rats, anticonvulsant effect of tocopherol and coenzyme Q10 were reported in a high-dose pilocarpine convulsion model (Tomé et al., 2010; Tawfik, 2011). If pretreatments interfere with seizure induction in chemically induced convulsions, for instance by modifying plasma or brain levels of pilocarpine or kainic acid, then reductions of oxidative stress are to be expected because (as shown in this study) oxidative stress follows seizure development and slowly subsides when seizures are interrupted. In our hands, however, no interference with the development and progress of SE was noted in any of our experimental groups. In other words, antioxidant effects measured in our study are not related to seizures, indicating a “true” antioxidant effect.

## 5 Conclusion

Summarizing, our data show that Li-Pilo-induced SE leads to a prominent increase of isoprostanes in rat hippocampus and a strong reduction of the GSH-GSSG ratio in the brain, indicating that seizures result in severe oxidative stress in the brain. Protein and DNA modifications were significant but much less prominent whereas the TBARS assay of MDA was not useful in our study. Oxidative stress subsides when seizures are terminated, a conclusion that could be drawn because our isoprostane-microdialysis model allows measurement of oxidative stress in real time. Among the six antioxidants tested, only pretreatment with vitamins C or E reduced isoprostane formation, attenuated the reduction of GSH-GSSG ratio and prevented increases of 8-OHdG and protein carbonyls during seizures. While vitamin E is known to terminate lipid peroxidation events, coenzyme Q10, an equally lipophilic compound, was inactive. Resveratrol, PBN and ebselen are all brain-permeable compounds, however, rapid metabolism or high hydrophilicity may have prevented their effectiveness in our SE model. Vitamin C, on the other hand, is highly hydrophilic and enters the brain via transporters. Its activity in our model may be due to the fact that vitamin C is capable of regenerating oxidized vitamin E. We conclude that high doses of vitamin C and E, given as a preventive measure and possibly in combination, may be useful to limit pathological consequences of recurrent seizure activity.

## Data Availability

The raw data supporting the conclusion of this article will be made available by the authors, without undue reservation.
