# Supplementary material for: Oxidative stress in rat brain during experimental status epilepticus: effect of antioxidants
Source: Front Pharmacol. 2023 Sep 12;14:1233184. doi: 10.3389/fphar.2023.1233184 (PMC10520702; doi:10.3389/fphar.2023.1233184)
Supplement: Supplementary file 1 [file Table1.DOCX]

Supplementary Material

Oxidative stress in rat brain during experimental status epilepticus: effect of antioxidants

# Marius Fuchs; Christian Viel; Alina Lehto; Helene Lau; Jochen Klein

# Institute of Pharmacology and Clinical Pharmacy, College of Pharmacy, Goethe University, Frankfurt am Main, Germany

# Correspondence: Jochen Klein, Institute of Pharmacology and Clinical Pharmacy, Goethe University Frankfurt, Max-von-Laue-Str.9, 60438 Frankfurt, Germany. Email: [klein@em.uni-frankfurt.de](mailto:klein@em.uni-frankfurt.de)

**
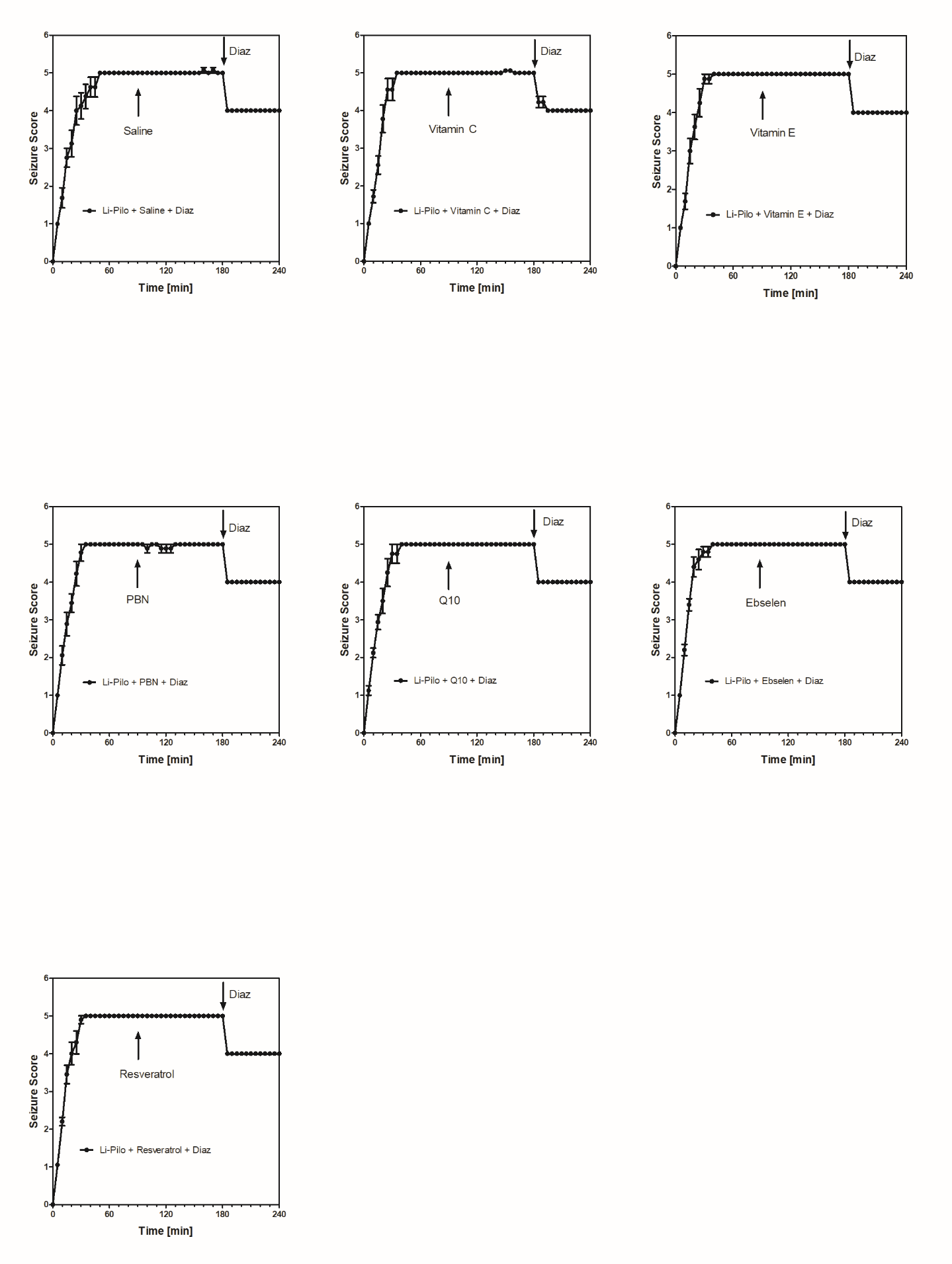
**Suppl. Fig. 1

Supplementary Fig. 1

Seizure score as measured by the modified Racine scale. Rats were pretreated with lithium chloride (127 mg/kg i.p.) and pilocarpine (30 mg/kg s.c.) was given 24 h later, at time zero (‘Li + Pilo’). Antioxidant or saline were injected 90 minutes after pilocarpine administration (indicated as arrow at time point 90 min). Seizures were terminated with diazepam 180 min after pilocarpine administration. Treatments: vitamin C; vitamin E; n-tert-butyl-α-phenylnitrone (PBN); coenzyme Q_10_ (Q10); ebselen and resveratrol. Injections of saline served as controls. Number of experiments (*N* = 8-10).


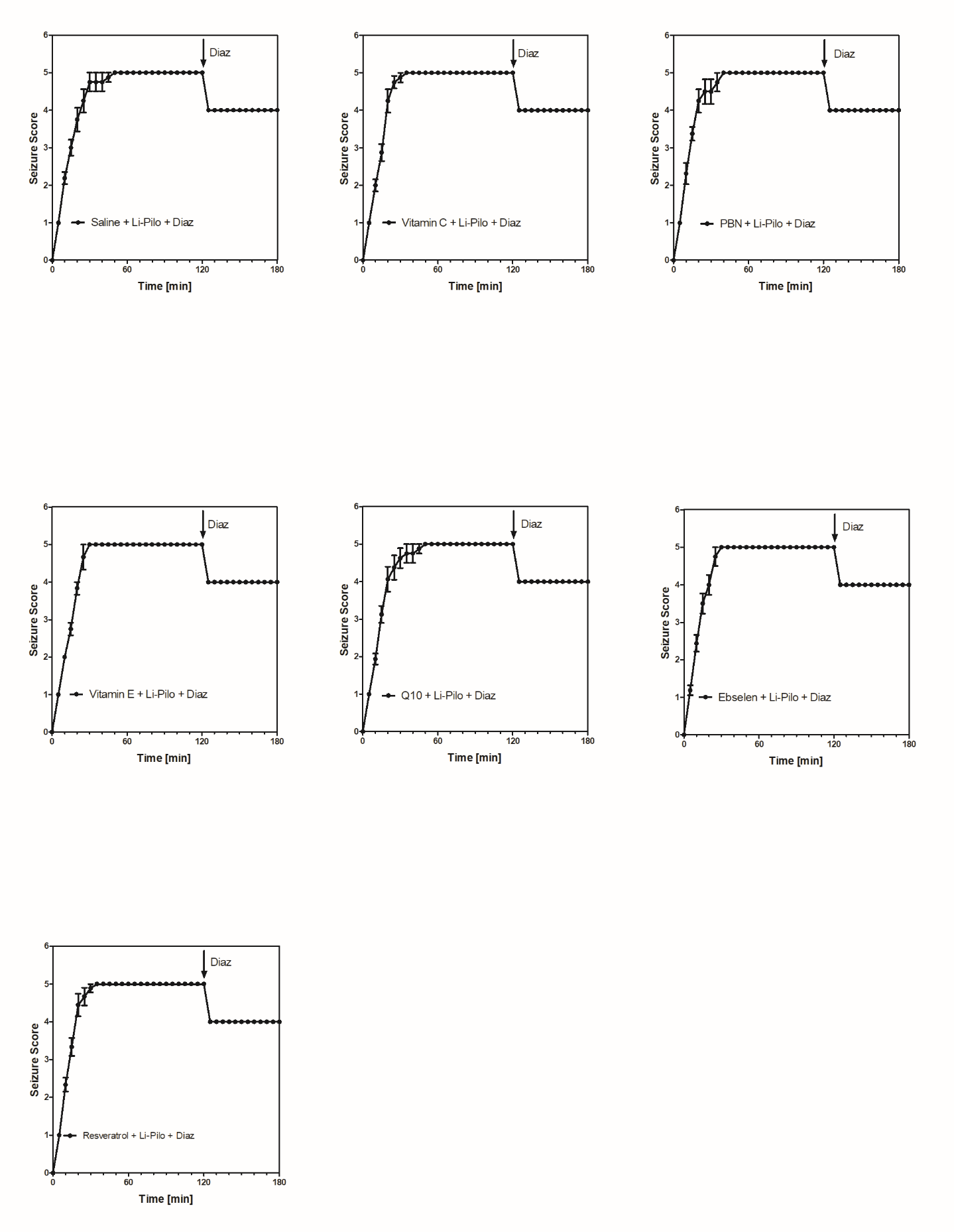
Suppl. Fig. 2

Supplementary Fig. 2

Seizure score as measured by the modified Racine scale. Rats were pretreated with lithium chloride (127 mg/kg i.p.) and pilocarpine (30 mg/kg s.c.) was given 24 h later, at time zero (‘Li + Pilo’). Rats received seven dosages of antioxidant or saline every twelve hours prior to seizure induction. Seizures were terminated with diazepam 120 min after pilocarpine administration. Treatments: vitamin C; vitamin E; n-tert-butyl-α-phenylnitrone (PBN); coenzyme Q_10_ (Q10); ebselen and resveratrol. Pretreatment with saline served as control. Number of experiments (*N* = 6-10).
